# Developmental Stage of Parasites Influences the Structure of Fish-Parasite Networks

**DOI:** 10.1371/journal.pone.0075710

**Published:** 2013-10-04

**Authors:** Sybelle Bellay, Edson Fontes de Oliveira, Mário Almeida-Neto, Dilermando Pereira Lima Junior, Ricardo Massato Takemoto, José Luis Luque

**Affiliations:** 1 Departamento de Ciências Biológicas, Programa de Pós-Graduação em Ecologia de Ambientes Aquáticos Continentais, Universidade Estadual de Maringá, Núcleo de Pesquisas em Limnologia, Ictiologia e Aqüicultura, Maringá, Paraná, Brazil; 2 Centro de Ciências Humanas e da Educação, Universidade Estadual do Norte do Paraná, Campus Jacarezinho, Grupo de Estudos e Pesquisa em Recursos Hídricos e Ecologia Aplicada, Jacarezinho, Paraná, Brazil; 3 Departamento de Engenharia Ambiental, Programa de Pós-Graduação em Engenharia Ambiental, Universidade Tecnológica Federal do Paraná, Campus Londrina, Londrina, Paraná, Brazil; 4 Instituto de Ciências Biológicas, Departamento de Ecologia, Universidade Federal de Goiás, Goiânia, Goiás, Brazil; 5 Departamento de Ciências Biológicas e da Saúde, Universidade Federal Mato Grosso, Campus Médio Araguaia, Pontal do Araguaia, Mato Grosso, Brazil; 6 Departamento de Parasitologia Animal, Universidade Federal Rural do Rio de Janeiro, Seropédica, Rio de Janeiro, Brazil; University of Toronto, Canada

## Abstract

Specialized interactions tend to be more common in systems that require strong reciprocal adaptation between species, such as those observed between parasites and hosts. Parasites exhibit a high diversity of species and life history strategies, presenting host specificity which increases the complexity of these antagonistic systems. However, most studies are limited to the description of interactions between a few parasite and host species, which restricts our understanding of these systems as a whole. We investigated the effect of the developmental stage of the parasite on the structure of 30 metazoan fish-parasite networks, with an emphasis on the specificity of the interactions, connectance and modularity. We assessed the functional role of each species in modular networks and its interactions within and among the modules according to the developmental stage (larval and adult) and taxonomic group of the parasites. We observed that most parasite and host species perform a few interactions but that parasites at the larval stage tended to be generalists, increasing the network connectivity within and among modules. The parasite groups did not differ among each other in the number of interactions within and among the modules when considering only species at the larval stage. However, the same groups of adult individuals differed from each other in their interaction patterns, which were related to variations in the degree of host specificity at this stage. Our results show that the interaction pattern of fishes with parasites, such as acanthocephalans, cestodes, digeneans and nematodes, is more closely associated with their developmental stage than their phylogenetic history. This finding corroborates the hypothesis that the life history of parasites results in adaptations that cross phylogenetic boundaries.

## Introduction

The application of network theory to the study of ecological interactions has significantly improved our understanding of the mechanisms generating community structure [1]–[3]. Indeed, the study of host-parasite interactions has been particularly favored by this theoretical and conceptual framework [4]–[8]. However, despite some recent advances in the elucidation of host-parasite networks, most studies have focused on the description of a few parasite and host species of ecological or commercial interest, which has resulted in the limited understanding of these systems as a whole. Host-parasite systems are very complex and include parasites with distinct life history strategies. For example, some fish parasites require intermediate hosts (i.e., parasites with a complex life cycle), whereas others need only a single host (i.e., parasites with a simple life cycle) [9], [10]. Furthermore, some fish may be parasitized by both larval and adult stages and hence be exposed to a wide variety of parasite species [9], [10].

Many patterns observed in ecological networks are related to the number of interactions (i.e., the degree) of each species and how these interactions are distributed throughout the network [11]. Specialized interactions tend to be more common in networks formed by ecological relationships that require a high degree of reciprocal adaptation between species [12]–[15], as is the case for most host-parasite networks in which the host selection varies widely among parasite taxa and between their larval and adult stages. These networks may reflect the strategies that parasites employ to reach the definitive hosts [3], [8], [16], [17].

Specialized host-parasite networks tend to be characterized by a low connectance (i.e., the number of interactions observed relative to the potential number of interactions in the network [18]); thus, these networks are expected to present a more modular structure than networks dominated by generalists [5], [6], [8]. A modular structure is characterized by groups of species that interact more with one another than with other species in the same network, thereby forming modules or compartments. There is evidence that the processes that create interaction patterns in ecological networks (e.g., phylogeny, coevolution, functional diversity and habitat structure) tend to be stronger within modules [6]–[8], [19], [20], and each species in a network has its own interaction pattern. In modular networks, these patterns characterize the functional role of species, i.e., how its interactions are distributed within the module to which it belongs and with respect to other modules [20]–[22]. These metrics may also be used to compare the interaction patterns between hosts and parasites and to compare developmental stages and taxonomic groups of parasites. This approach allows the identification of which species or groups are most important to the structure of the entire network, for example, by connecting different modules to each other.

The main objective of the present study was to assess the effect of the developmental stage of parasites on the structure of the interactions in fish-metazoan parasite networks. First, we evaluated the proportion of parasites and hosts in the networks, verifying both the host susceptibility and parasite specificity. Second, we analyzed the relationship between connectance and species richness in the studied networks and the proportions of parasites at the larval and adult stages. Third, we assessed the interaction patterns within and among the modules of modular networks with regard to different developmental stages and taxonomic groups of parasites. We expected (a) a predominance of specialized interactions in the host-parasite networks, resulting in a low connectance, a pattern that should occur mainly in species-rich networks because, with an increase in richness, more hosts tend to enter into the network with their specific parasites. However, a greater proportion of larval stage parasites in the network should exhibit an increase in connectance because, in general, larval stages tend to interact with more species of fish hosts than adult parasites. We also expected (b) differences in the distribution of interactions within and among the modules of parasites at the larval and adult stages, independent of the phylogenetic group, because their requirements involve different ecological resources (e.g., host species, infection sites and nutrients). Therefore, a low co-occurrence between parasites at the larval and adult stages of the same species is expected in modules composed of species with both developmental stages.

## Materials and Methods

### Database

We analyzed data on 30 fish-parasite networks compiled from the literature (see Dataset S1). We organized the data as binary matrices with parasites in the rows and hosts in the columns. When both the larval and adult stages of a parasite were found in the same network, we regarded them as functionally different species [4]. In this analysis, we included only networks with metazoan parasites due to the scarcity of information regarding other groups (i.e., bacteria, fungi and protozoa).

### Network Descriptors

For each network, we calculated its connectance and the proportion of parasites at the larval and adult stages in relation to the total number of species in the network. To test the existence of modules in the host-parasite networks, we used a simulated annealing algorithm in NETCARTO [21], [22]. We then calculated a modularity index (*M*) for each network using the following formula [21], [22]: 
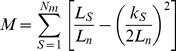
where *N_m_* is the number of modules in the network, *L_S_* is the number of interactions among all species within the module *s*, *L_n_* is the number of interactions in the network, and *k_S_* is the sum of the degree (i.e., total number of interactions) of all species in the module *s*. A value of *M* = 0 indicates interactions with random distribution, whereas a value close to *M* = 1 indicates a strongly modular network [23].

The randomized matrices that are generated directly by the software NETCARTO consider the network as unipartite, and the software does not provide the recommended null model (CE null model) [21], [22]. Therefore, considering the bipartite structure of the networks, we used a function developed by Nadson RS da Silva for R [24] to generate 1,000 randomized bipartite matrices with the CE null model for each network analyzed to estimate the significance of *M*. This null model is more conservative than those that simply relocate the interactions randomly for all cells of the matrix [1], [25], and it tends to conserve most of the heterogeneity in the distribution pattern of the interactions among species. In addition, this model reduces the type I error in the detection of patterns in networks with low connectance [26], as in the case of host-parasite networks. We calculated *M* for each of the 1,000 matrices in NETCARTO using a Fortran code that automates the calculation and compilation of the *M* values (provided by Flávia MD Marquitti; [27]). We estimated the significance of *M* (p value) for each matrix by counting the number of random matrices that showed an *M* value equal to or higher than the *M* value of the real matrix and dividing it by the total number of random matrices. We defined p<0.001 when no random matrix showed an *M* value higher than that of the real matrix [27].

### Functional Roles

For modular networks, the functional role of a species *i* is defined by its *z* value (i.e., the standardized number of interactions of species *i* within its module *s*). 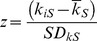
and *c* value (i.e., the connectivity of species *i* among modules; also known as the participation coefficient *P* [21], [22]):



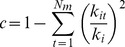
where *k_is_* is the number of interactions of species *i* with other species within its module *s*; *k_s_* and *SD_ks_* are the average and standard deviation of *k* within the module, considering all the species in *s*, respectively; *N_m_* is the number of modules in the network; *k_it_* is the number of interactions of species *i* with the other species of module *t* (including species *i*’s own module); and *k_i_* is the total number of interactions of species *i* in the network [20]–[22].

We calculated the values of *z* considering the bipartite structure of the networks, assessing the average and standard deviation of the interactions for hosts and parasites separately. For species from modules with *SD_ks_* = 0, we assumed *z* = 0 because the species did not have a connectivity within the module that was higher or lower than the average value of the module [8]. We obtained the values of species connectivity among the modules in NETCARTO because they do not differ between unipartite and bipartite networks [8]. If all the interactions of a species occur within its module, then *c* = 0; however, if all the interactions are uniformly distributed among the modules, the *c* value will tend toward 1 [20]. Based on the *z* and *c* values calculated from each matrix, we classified the species as follows: peripheral (*z* ≤2.5 and *c* ≤0.62, i.e., with a few interactions with other species); connector (z ≤2.5 and *c*>0.62, i.e., connects several modules to each other); module hub (*z*>2.5 and *c* ≤0.62, i.e., has several interactions within its module); or network hub (*z*>2.5 and *c*>0.62, i.e., the species is a connector and has several interactions in the module) [20].

### Data Analysis

We calculated the susceptibility of a host species based on the proportion of parasite species present in its network and the specificity of parasites based on the proportion of host species with which each parasite interacted. We tested for relationships between the connectance and species richness and between the connectance and proportion of parasite species at the larval and adult stages using a Pearson correlation. Whenever necessary, the data were log-transformed to meet the assumption of normality.

We tested for differences between the *z* and *c* values of the host and parasite species and of the larval and adult parasites, considering all the hosts and only those parasites identified to a specific level (genus and specific epithet). Only parasites with a defined species were used in the analyses to allow comparisons of species among the networks and to evaluate the possible variations regarding the functional roles of these species. In addition, we tested for differences in the *z* values of the host and parasite species considered less or more interactive (negative and positive *z* values, respectively [8]). Lastly, to control for the effect of the high number of parasites with *c* = 0, we compared the connectivity of the hosts and parasites among the modules considering only values of *c*>0. We evaluated the differences in the *z* and *c* values between the parasites at the larval and adult stages, first considering all the groups of parasites studied and then evaluating only those groups with both stages in the networks. We analyzed the developmental stage distributions among the modules in the network formed by those species with both stages and differences between their *z* and *c* values. We performed the Wilcoxon test for paired samples to compare the *z* and *c* values using the mean values observed for each network. All these statistical analyses were conducted in Statistica 7.0 [28].

We also assessed the *z* and *c* values among taxonomic groups with parasites at the larval and adult stages, considering each stage separately. For these analyses, we used PERMANOVA [29] available in the vegan package for R [26], applying Euclidean distance to construct the dissimilarity matrix and controlling the relationships among the groups based on their phylogenetic histories (Phylum level). We performed 1,000 randomizations for the significance test.

## Results

**Figure 1:**
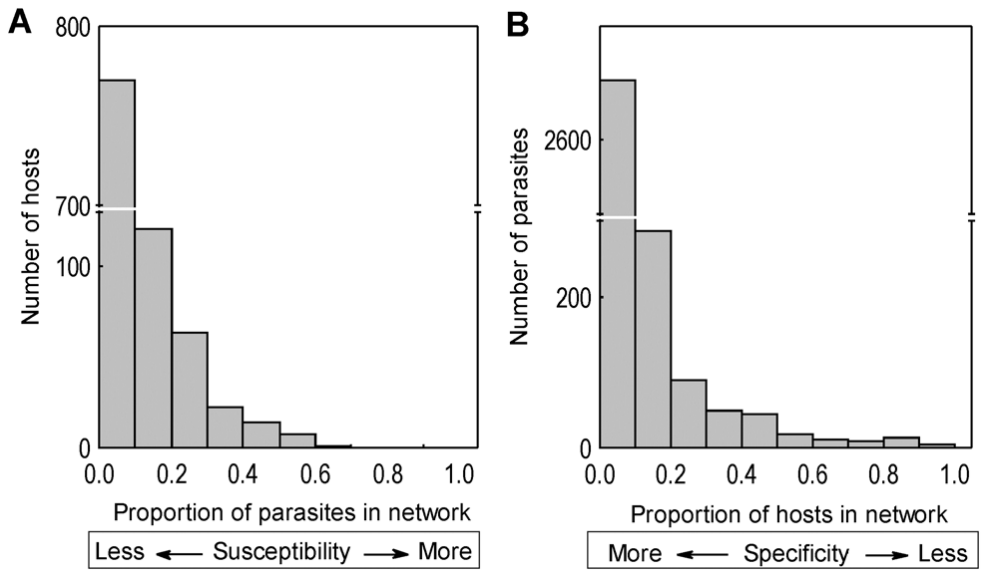
Interspecific interactions in 30 fish-parasite networks. When we assessed (a) the susceptibility of the hosts in relation to the proportion of parasite species in networks and (b) the specificity of the parasites in relation to the proportion of host species in networks, we observed that most parasites interact with a few fish species and that the hosts typically have a poor parasitic fauna.

**Figure 2:**
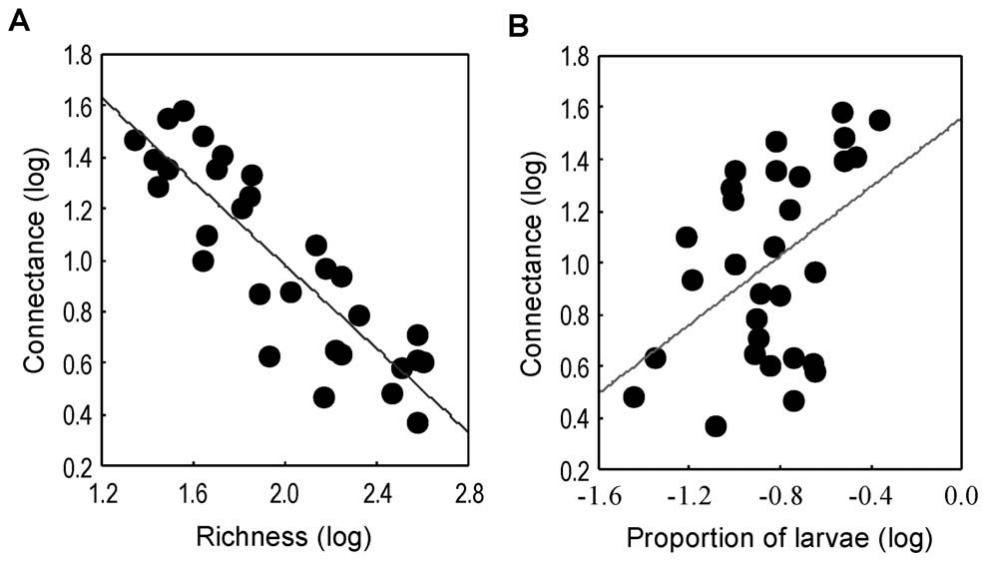
Correlations between the parameters calculated for 30 fish-parasite networks. The relationship (a) between species richness and connectance, showing that species-rich networks have a low connectance, and (b) between the proportion of parasite species at the larval stage and connectance, evidencing the contribution of this stage to an increase in connectance.

The descriptors of each network are presented in Table S1. The general structure of the networks analyzed revealed a tendency of a few hosts to have a high susceptibility to parasitism (Fig. 1A); similarly, a low number of parasites was found in several hosts (Fig. 1B). As expected due to the low host susceptibility and high parasite specificity, the values of connectance (%) observed in the networks were relatively low (average = 13.81; SD = 10.71). This parameter was negatively correlated with the total number of species (r = −0.87; p<0.001; Fig. 2A) and positively correlated with the proportion of parasite species at the larval stage in the networks (r = 0.46, p = 0.01; Fig. 2B). Connectance was not correlated with the proportion of adults in the networks, though a non-significant negative tendency was observed (r = −0.27, p = 0.15).

**Figure 3:**
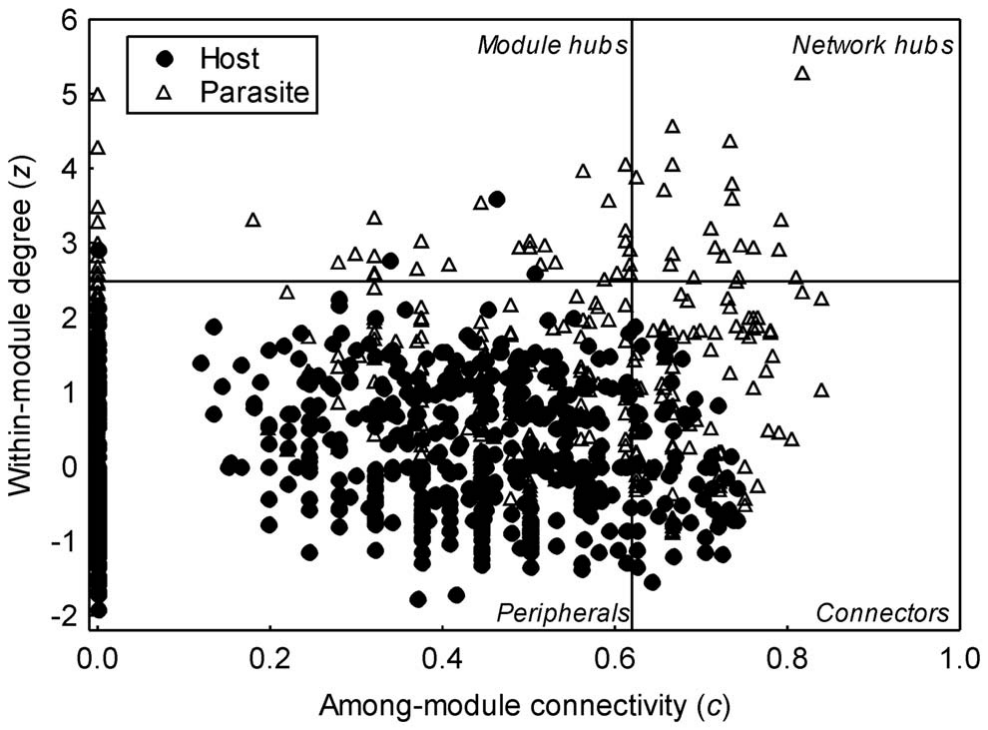
Functional roles of different species in 20 fish-parasite networks with a modular structure. According to the *z* and *c* values, we observed a large number of peripheral species and the role of parasites as connectors within and among the modules.

Twenty networks were significantly modular (average *M* = 0.59; SD = 0.12). Considering only parasites with a defined species, most taxa in these modular networks were classified as peripheral (765 hosts and 1,881 parasites), followed by connectors (59 hosts and 94 parasites), module hubs (four hosts and 39 parasites), and network hubs (20 parasites) (Fig. 3). For species recorded in more than one network (see Dataset S1), we found that they showed the same functional role in different networks in 82.20% of the cases. All species that showed changes among the networks had a functional role that was peripheral in at least one of the networks. A separate evaluation according to the stage of development revealed changes in the functional role of 34.78% and 14.82% of species at the larval and adult stages, respectively. The hosts and parasites did not differ with regard to their *z* values (T Wilcoxon = 93; p = 0.65). However, when the *z* values were compared separately between less interactive (*z* negative values; 405 hosts and 1,190 parasites) and more interactive species (*z* positive values; 286 hosts and 574 parasites), we observed that, in both cases, the parasites showed a higher number of interactions within their modules than the hosts (*z* of less interactive species: T Wilcoxon = 30, p = 0.005; *z* of more interactive species: T Wilcoxon = 31, p = 0.005). In contrast, the hosts showed higher *c* values (T Wilcoxon = 7; p<0.001) than the parasites. Considering only those hosts and parasites with *c* values>0, the results (T Wilcoxon = 39; p = 0.013) corroborated the tendency observed in Fig. 3, demonstrating the contribution of the parasites to the connectivity among modules.

**Figure 4:**
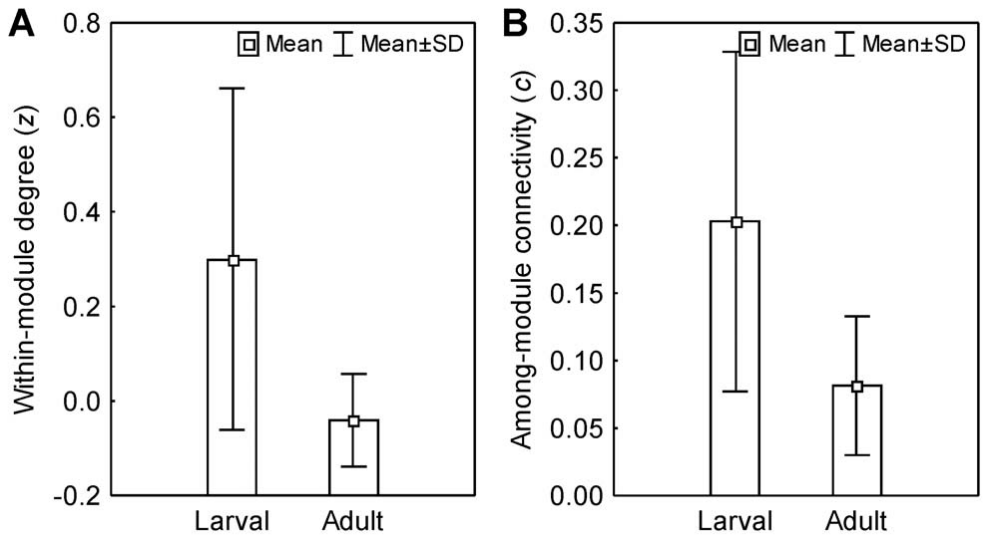
Distribution of interactions within and among modules of larval and adult parasites in 20 fish-parasite networks with a modular structure. The differences between the *z* (a) and *c* (b) values of the parasite larvae and adults show a tendency toward generalism in the interactions performed by parasites at the larval stage within and among modules.

Considering only parasites with a defined species, 219 larvae and 1,662 adults were classified as peripherals, 32 larvae and 62 adults as connectors, nine larvae and 30 adults as module hubs, and eight larvae and 12 adults as network hubs. Considering all the taxonomic groups of the parasites studied, the larvae presented higher values of *z* (T Wilcoxon = 24, p = 0.002; Fig. 4A) and *c* (T Wilcoxon = 17; p = 0.001; Fig. 4B) than the adults in the networks. The larval stages also presented higher values of *z* (T Wilcoxon = 41, p = 0.016) and *c* (T Wilcoxon = 21; p = 0.004) when possible differences in the adult stages were tested, evaluating only taxa with species in both stages in the network (i.e., Digenea and Cestoda both Platyhelminthes, Acanthocephala and Nematoda). However, when we evaluated the values of *z* and *c* for parasite species with larval and adult stages present in the same network, which do not co-occur in the same module in 72% of the cases, we observed higher values of *z* for the adult parasites (T Wilcoxon = 1181; p = 0.016) and no significant differences in the *c* values (T Wilcoxon = 794; p = 0.371) between these two stages.

In Table 1, we show the mean values of *z* and *c* for the taxonomic groups of the studied parasites, considering these groups separately according to the stage of development. The taxonomic groups of parasites with larval and adult stages in the networks did not differ in their distribution of interactions within and among the modules, when considering parasites at the larval stage (*z*: F = 1.52, p = 0.22; *c*: F = 0.63, p = 0.62). However, when considering only adult parasites, significant differences were observed in their distribution of interactions within (*z*: F = 14.99, p<0.001) and among modules (*c*: F = 40.15, p<0.001).

**Table 1:** Values of *z* and *c* of 13 groups of metazoan parasites at larval and adult stages in 20 fish-parasite networks with a modular structure.

	Larval		Adult	
Group			z	c
Acanthocephala	0.350	0.198	0.560	0.296
Aspidobothrea			−0.180	0.208
Branchiura			0.032	0.176
Cestoda	0.128	0.249	−0.074	0.068
Copepoda			0.276	0.138
Digenea	0.475	0.272	−0.007	0.069
Hirudinea			−0.051	0.146
Isopoda			−0.180	0
Monogenea			−0.258	0.012
Myxosporea			−0.335	0.021
Nematoda	0.237	0.226	0.137	0.131
Mollusca	−0.155	0.166		
Pentastomida	0	0		

## Discussion

The level of reciprocal adaptation between species in an ecological network determines the basis of its architecture and its patterns of interspecific connectivity [30]. In host-parasite networks, the host body provides a microhabitat for parasite species of different lineages and with different life history strategies (e.g., endoparasites or ectoparasites and parasites with simple or complex life cycles). Therefore, due to the different ways that parasites may exploit a resource, we could expect a high number of parasites to be associated with each host species. However, these microhabitats are limited environments, and even organisms that are highly susceptible to parasitism do not harbor all the parasites found in a network. In addition, the host exhibits biological (e.g., body size and immune system) and ecological (e.g., feeding habit and exposure to infecting forms resulting from behavioral activities) constraints that prevent interactions with a large number of parasite species. These constraints contribute to the strong negative relationship between richness and connectance observed in the present study, as these limitations reduced the number of parasite species per host. Other studies have also shown this negative relationship between species richness and connectance in host-parasite networks (see [31], [32]), indicating that this is a typical pattern for this type of antagonistic interaction network.

In addition to the host characteristics, the high level of parasite specialization, resulting from the evolutionary history of each species, contributes strongly to the formation of modular structures in host-parasite networks. Hence, the low number of interactions performed by most parasites most likely reflects a trade-off between the benefit of having alternative hosts and the cost of developing adaptations that allow the parasites to remain in the hosts for long periods. Thus, parasites obtain a balanced diet and avoid enemies, maximizing the use of the host species that provide them with the best conditions for the development of a stable interaction [12], [33].

Considering the interaction patterns within and among the modules in a network, most species was peripheral. A few species that occurred in more than one network showed variations in their functional role, with the highest variation observed among species at the larval stage. The high host specificity among parasites explains the high frequency of peripheral parasites and thus the low variation in the functional roles of species in different networks. However, the variation in the functional roles of a species among networks is a reflection of the complexity of the interactions, which are partly under the influence of the structure and dynamics of each environment [19], [34]. The major change among the functional roles of species at the larval stage may be a reflection of processes aimed at optimizing the access to the definitive host according to the characteristics of the environment. In this context, variations in the population sizes of fish or invertebrates that act as the first intermediate host in several cycles, for example, may benefit from a broader or more restricted distribution among the possible species of hosts for parasites at the larval stage. However, some hosts and parasites showed an important role in network connectivity, mainly the parasites at the larval stage, because larvae are usually more generalistic than adults [35], which tend to increase network connectivity. In the present study, only the parasite species that showed several interactions within and among the modules and were considered network hubs.

Our study reveals the trend of parasite species at the larval stage being more generalist than species at the adult stage in fish-parasite networks. In fish, adult parasites include species with life cycles that are simple or complex and can be endo- or ectoparasites. In particular, a high host specificity is known in the case of monogenean ectoparasites and may reflect the type of infection strategy of those parasites with a simple cycle [36], which contributes to the parasite species at the adult stage being more specific than at the larval stage. However, the influence of ectoparasites in differentiating between larvae and adults is discounted when evaluating only groups of parasites with both stages in the networks.

Accessibility to the definitive hosts may be related to the evolutionary strategies of parasites, with species at the larval stage being less sensitive than adults to the morpho-physiological variations observed among host species of a particular fish habitat; additionally, some species at the adult stage are actually more dependent on the particular characteristics of their host species. However, exceptions to the general pattern with many interactions for species at the larval stage may be observed in these networks. For adults that co-occur with the larval stage of their own species in the networks, the trend of having a higher number of interactions may be an indication that the distribution of the larvae of these species is guided by adaptations to certain species of fish as intermediate hosts that commonly serve as more accessible prey to several species of fish that are the definitive host.

The low co-occurrence within the modules of the larval and adult stages in those species of parasites with both developmental stages in the networks reinforces the evidence that these stages have distinct functions in the networks, as previously been proposed [4], [6], [7]. The organization of host-parasite networks in modules reflects the similarity of the resources provided by some hosts [6], [8]. Hence, when the parasite co-occurrence pattern is assessed within each module, it tends to not differ from what would be expected by chance [6]. Phylogenetically close hosts tend to belong to the same modules, whereas parasites of several lineages may converge and interact with hosts of different modules [6]–[8]. Parasite species that are phylogenetically distant may occur in the same modules, but this functional convergence does not indicate that they have similar interaction patterns in a network. The phylogeny of parasites has been indicated to be an important characteristic that establishes the role that the species perform in the networks [19]. For example, in the present study, monogeneans showed low *z* and *c* values compared to other groups (Table 1), corroborating the hypothesis of the high host specificity reported for this group [17], [37].

In some cases, similarity in the interaction patterns may be observed in different taxonomic groups, but only at particular developmental stages of the parasite. The *z* and *c* values of the taxonomic groups did not differ from each other when the parasite species were at the larval stage, but the same taxonomic groups showed different interaction patterns at the adult stage. This finding reinforces that parasite specificity, as reflected here in the interaction patterns of some groups (e.g., acanthocephalans, cestodes, digeneans and nematodes), within and among modules is more closely related to the life stage than to the taxonomic group. Indeed, parasite life histories produce adaptations that cross phylogenetic boundaries and may promote both convergent and divergent evolution [12], [38].

Host-parasite interactions depend on mechanisms that provide the parasites with a better chance to complete their life cycle [17]. These adaptations, mainly those observed in generalistic parasites, are not necessarily related to particular co-evolutionary processes [39] and may be exemplified by the low specificity of some parasites to an intermediate host that is prey to the definitive host. In this system of trophic transmission, the parasite may have a higher chance to reach the definitive host if it is widely distributed among the prey of this host [40]. These intermediate hosts may have very distinct parasite faunas and be distributed in different modules, which may be connected to each other by generalistic parasites.

Therefore, for some species of parasites, occurring in an intermediate host that is often preyed on by the definitive host, as previously mentioned, is more advantageous according to the size and abundance of these prey in the environment and the co-occurrence between predator and prey habitats [7], [41], [42]. This may be a pattern for autochthonous parasites (i.e., parasites that reach maturity in hosts in the same environment as their larvae; the aquatic environment in the case of fish-parasite interactions) in which a smaller number of interactions with fish intermediate hosts than with definitive hosts was observed for the parasites with both developmental stages in the networks. The variation in the number of trophic interactions between different types of definitive hosts (i.e., fish and birds) suggests some effect on the connectivity pattern of parasites [19]. However, more studies are necessary to clarify the possible patterns that distinguish the generality of the interactions between the larvae of autochthonous and allochthonous parasites (i.e., parasites with larval and adult stages in different environments, e.g., in terrestrial and aquatic environments). After demonstrating the importance of different parasite developmental stages in fish-parasite networks characterized by a high interaction specificity, further studies may assess which factors are responsible for increases or decreases in the number of parasites at the larval stage in these networks.

## Supporting Information

Table S1 **Descriptors measured for 30 fish-parasite networks.**
(DOC)

Dataset S1 **Matrices of 30 fish-parasite networks and values of** ***z*** **and** ***c*** **in 20 fish-parasite networks with a modular structure.**
(XLS)

## References

[1] Bascompte J, Jordano P, Melian CJ, Olesen JM (2003) The nested assembly of plant–animal mutualistic networks. Proc Natl Acad Sci U S A 100: 9383–9387. 12881488 10.1073/pnas.1633576100 PMC170927

[2] Proulx RS, Promislow DEL, Phillips PC (2005) Network thinking in ecology and evolution. Trends Ecol Evol 460: 345–353. 10.1016/j.tree.2005.04.004 16701391

[3] Poulin R (2010) Network analysis shining light on parasite ecology and diversity. Trends Parasitol 26: 492–498. 20561821 10.1016/j.pt.2010.05.008

[4] Vázquez DP, Poulin R, Krasnov BR, Shenbrot GI (2005) Species abundance and the distribution of specialization in host–parasite interaction networks. J Anim Ecol 74: 946–955.

[5] Fortuna MA, Stouffer DB, Olesen JM, Jordano P, Mouillot D, et al (2010) Nestedness versus modularity in ecological networks: two sides of the same coin? J Anim Ecol 79: 811–817. 20374411 10.1111/j.1365-2656.2010.01688.x

[6] Bellay S, Lima Jr DP, Takemoto RM, Luque JL (2011) A host-endoparasite network of Neotropical marine fish: are there organizational patterns? Parasitology 138: 1945–1952. 21854678 10.1017/S0031182011001314

[7] Lima Jr DP, Giacomini HC, Takemoto RM, Agostinho AA, Bini LM (2012) Patterns of interactions of a large fish–parasite network in a tropical floodplain. J Anim Ecol 81: 905–913. 22339475 10.1111/j.1365-2656.2012.01967.x

[8] Krasnov BR, Fortuna MA, Mouillot D, Khokhlova IS, Shenbrot GI, et al (2012) Phylogenetic signal in module composition and species connectivity in compartmentalized host-parasite networks. Am Nat 179: 501–511. 22437179 10.1086/664612

[9] Thatcher VE (2006) Aquatic biodiversity in Latin America: Amazon Fish Parasites Volume 1, 2nd edition. Sofia: Pensoft. 496 p.

[10] Woo PTK (2006) Fish diseases and disorders, protozoan and metazoan infections: Volume 1, 2nd edition. Cambridge: CAB International. 791 p.

[11] Lewinsohn TM, Prado PI (2006) Structure in plant-animal interaction assemblages. Oikos 113: 174–184.

[12] Thompson JN (1994) The coevolutionary process. Chicago: University of Chicago Press. 376 p.

[13] Thompson JN (2005) The geographic mosaic of coevolution. Chicago: University of Chicago Press. 400 p.

[14] Blüthgen N, Frund J, Vázquez DP, Menzel F (2008) What do interaction network metrics tell us about specialization and biological traits? Ecology 89: 3387–3399. 19137945 10.1890/07-2121.1

[15] Guimarães PR, Rico-Gray V, Oliveira PS, Izzo TJ, Dos Reis SF, et al (2007) Interaction intimacy affects structure and coevolutionary dynamics in mutualistic networks. Curr Biol 17: 1797–1803. 17949981 10.1016/j.cub.2007.09.059

[16] Palm HW, Caira JN (2008) Host specificity of adult versus larval cestodes of the elasmobranch tapeworm order Trypanorhyncha. Int J Parasitol 38: 381–388. 17950740 10.1016/j.ijpara.2007.08.011

[17] Poulin R (1992) Determinants of host-specificity in parasites of freshwater fishes. Int J Parasitol 22: 753–758. 1428509 10.1016/0020-7519(92)90124-4

[18] Pimm SL (1982) Food webs. London: Chapman & Hall. 219 p.

[19] Poulin R, Krasnov BR, Pilosof S, Thieltges DW (2013) Phylogeny determines the role of helminth parasites in intertidal food webs. J Anim Ecol. DOI: 10.1111/1365–2656.12101. 10.1111/1365-2656.12101 23800281

[20] Olesen JM, Bascompte J, Dupont YL, Jordano P (2007) The modularity of pollination networks. Proc Natl Acad Sci U S A 104: 19891–19896. 18056808 10.1073/pnas.0706375104 PMC2148393

[21] Guimerà R, Amaral LAN (2005) Functional cartography of complex metabolic networks. Nature 433: 895–900. 15729348 10.1038/nature03288 PMC2175124

[22] Guimerà R, Amaral LAN (2005) Cartography of complex networks: modules and universal roles. J Stat Mech P02001. 10.1088/1742-5468/2005/02/P02001 PMC2151742 18159217

[23] Newman MEJ, Girvan M (2003) Finding and evaluating community structure in networks. Phys Rev E Stat Nonlin Soft Matter Phys 69: 026113. 10.1103/PhysRevE.69.026113 14995526

[24] R Development Core Team (2011) R: A language and environment for statistical computing. R Foundation for Statistical Computing, Vienna, Austria. ISBN 3–900051–07–0. Available: http://www.R-project.org. Accessed 9 December 2012.

[25] Guimarães Jr PR, Guimarães PR (2006) Improving the analyses of nestedness for large sets of matrices. Environ Modell Softw 21: 1512–1513.

[26] Podani J, Schmera D (2012) A comparative evaluation of pairwise nestedness measures. Ecography 35: 889–900.

[27] Mello MAR, Marquitti FMD, Guimarães Jr PR, Kalko EKV, Jordano P, et al (2011) The missing part of seed dispersal networks: structure and robustness of bat-fruit interactions. PLoS One 6: e17395. 21386981 10.1371/journal.pone.0017395 PMC3046224

[28] STATSOFT Inc. (2005) Statistica (data analysis software system) version 7.1. Tulsa, USA. Available: www.statsoft.com. Accessed 9 December 2012.

[29] Anderson MJ (2001) A new method for non-parametric multivariate analysis of variance. Austral Ecol 26: 32–46.

[30] Jordano P, Bascompte J, Olesen JM (2006) The ecological consequences of complex topology and nested structure in pollination webs. In: Waser NM, Ollerton J, editors. Plant-Pollinator Interactions: From Specialization to Generalization. Chicago: University of Chicago Press. 173–199.

[31] Mouillot D, Krasnov BR, Shenbrot GI, Poulin R (2008) Connectance and parasite diet breadth in flea-mammal webs. Ecography 31: 16–20.

[32] Poulin R (2007) Are there general laws in parasite ecology? Parasitology 134: 763–776. 17234043 10.1017/S0031182006002150

[33] Poulin R (1998) Evolutionary ecology of parasites, 1st edition. London: Chapman & Hall. 212 p.

[34] Khan RA (2012) Host-parasite interactions in some fish species. J Parasitol Res 2012: ID 237280. 10.1155/2012/237280 PMC3415075 22900144

[35] Esch GW, Fernández JC (1993) A Functional Biology of Parasitism. London: Chapman & Hall. 337 p.

[36] Dobson A, Lafferty KD, Kuris AM, Hechinger RF, Jetz W (2008) Homage to Linnaeus: how many parasites? How many hosts? Proc Natl Acad Sci U S A 105 Suppl: 11482–11489 10.1073/pnas.0803232105 PMC2556407 18695218

[37] Whittington ID, Kearn GC (2011) Hatching strategies in monogenean (Platyhelminth) parasites that facilitate host infection. Integr Comp Biol 51: 91–99. 21558179 10.1093/icb/icr003

[38] Price PW (1980) Evolutionary biology of parasites. Princeton: Princeton University Press. 237 p.

[39] Barker SC (1991) Evolution of host-parasite associations among species of lice and rock-wallabies: coevolution? Int J Parasitol 21: 497–501. 1743847 10.1016/0020-7519(91)90053-a

[40] Choisy M, Brown SP, Lafferty KD, Thomas F (2003) Evolution of trophic transmission in parasites: why add intermediate hosts? Am Nat 162: 172–181. 12858262 10.1086/375681

[41] Bozza AN, Hahn NS (2010) Uso de recursos alimentares por peixes imaturos e adultos de espécies piscívoras em uma planície de inundação neotropical. Biota Neotrop 10: 217–226.

[42] Luque JL, Poulin R (2004) Use of fish as intermediate hosts by helminth parasites: A comparative analysis. Acta Parasitol 49: 353–361.

