# Supplementary material for: Developmental Stage of Parasites Influences the Structure of Fish-Parasite Networks
Source: PLoS One. 2013 Oct 4;8(10):e75710. doi: 10.1371/journal.pone.0075710 (PMC3790841; doi:10.1371/journal.pone.0075710)
Supplement: Table S1 — Descriptors measured for 30 fish-parasite networks. (DOC) [file pone.0075710.s001.doc]

**Table**

**Table S1. Descriptors measured for 30 fish-parasite networks.**

| **Network** | ***S*** | ***H*** | ***Pa*** | ***La*** | ***Ad*** | ***I*** | ***C*** | ***M*** | **p** | ***Reference*** |
| --- | --- | --- | --- | --- | --- | --- | --- | --- | --- | --- |
| **1- Mid Paraná River, Argentina** | 147 | 54 | 93 | 0.08 | 0.55 | 146 | 2.91 | 0.81 | <0.001 | [1] |
| **2- Floodplain of Upper Paraná River, Brazil** | 376 | 65 | 311 | 0.09 | 0.72 | 472 | 2.33 | 0.73 | <0.001 | [2,3] |
| **3- Smallwood Reservoir, Canada** | 31 | 6 | 25 | 0.06 | 0.74 | 53 | 35.33 | 0.32 | 0.39 | [4] |
| **4- Parsnip River, Canada** | 70 | 17 | 53 | 0.1 | 0.65 | 158 | 17.54 | 0.44 | 0.003 | [5] |
| **5- McGregor River, Canada** | 65 | 14 | 51 | 0.12 | 0.66 | 114 | 15.97 | 0.45 | 0.11 | [5] |
| **6- Lake of the Woods, Canada** | 175 | 31 | 144 | 0.13 | 0.69 | 384 | 8.6 | 0.48 | <0.001 | [6] |
| **7- Cold Lake, Canada** | 50 | 10 | 40 | 0.16 | 0.64 | 91 | 22.75 | 0.44 | 0.02 | [7] |
| **8- Aishihik Lake, Canada** | 36 | 7 | 29 | 0.22 | 0.58 | 78 | 38.42 | 0.27 | 0.48 | [8] |
| **9- Coastal Waters of Rio de Janeiro, Brazil** | 378 | 39 | 339 | 0.14 | 0.75 | 678 | 5.13 | 0.61 | <0.001 | [9] |
| **10- Little Colorado River, USA** | 31 | 11 | 20 | 0.23 | 0.41 | 50 | 22.727 | 0.38 | 0.43 | [10] |
| **11- Lake Michigan, Canada-USA** | 165 | 45 | 120 | 0.22 | 0.50 | 240 | 4.44 | 0.63 | 0.001 | [11] |
| **12- Lake Superior, Canada-USA** | 211 | 36 | 175 | 0.19 | 0.63 | 381 | 6.05 | 0.58 | <0.001 | [11] |
| **13- Guandu River, Brazil** | 107 | 22 | 85 | 0.15 | 0.64 | 141 | 7.54 | 0.64 | <0.001 | [12] |
| **14- Łebsko Lagoon, Poland** | 22 | 8 | 14 | 0.18 | 0.45 | 33 | 29.46 | 0.4 | 0.16 | [13] |
| **15- Zarivar Lake, Iran** | 28 | 8 | 20 | 0.04 | 0.67 | 31 | 19.37 | 0.52 | 0.13 | [14] |
| **16- St. Mary’s River, Canada-USA** | 78 | 26 | 52 | 0.31 | 0.35 | 100 | 7.4 | 0.6 | 0.05 | [11] |
| **17- Lake Huron, Canada-USA** | 382 | 79 | 303 | 0.18 | 0.61 | 977 | 4.08 | 0.49 | <0.001 | [11] |
| **18- Lake St. Clair, Canada-USA** | 44 | 13 | 31 | 0.43 | 0.27 | 40 | 9.92 | 0.67 | 0.03 | [11] |
| **19- Lake Erie, Canada-USA** | 399 | 91 | 308 | 0.17 | 0.59 | 1117 | 3.98 | 0.46 | <0.001 | [11] |
| **20- Lake Ontario, Canada-USA** | 321 | 61 | 260 | 0.15 | 0.65 | 602 | 3.79 | 0.58 | <0.001 | [11] |
| **21- Devils Lake, USA** | 27 | 6 | 21 | 0.3 | 0.48 | 31 | 24.60 | 0.51 | 0.03 | [15] |
| **22- Bay of Bengal, Bangladesh** | 86 | 37 | 49 | 0.13 | 0.44 | 77 | 4.25 | 0.83 | <0.001 | [16] |
| **23- Hidvégi Lake, Hungary** | 46 | 12 | 34 | 0.13 | 0.60 | 51 | 12.5 | 0.62 | 0.01 | [17] |
| **24- Gulf of Riga, Latvia** | 150 | 52 | 98 | 0.15 | 0.50 | 469 | 9.2 | 0.42 | <0.001 | [18] |
| **25- Lake Alüksnes, Latvia** | 72 | 48 | 24 | 0.09 | 0.23 | 247 | 21.44 | 0.3 | 0.19 | [18] |
| **26- Lake Raznas, Latvia** | 137 | 48 | 89 | 0.1 | 0.54 | 490 | 11.47 | 0.33 | 0.11 | [18] |
| **27- Tres Palos Lagoon, Mexico** | 53 | 13 | 40 | 0.3 | 0.45 | 132 | 25.38 | 0.28 | 0.87 | [19] |
| **28- Mekong River Delta, Vietnam** | 178 | 52 | 126 | 0.04 | 0.66 | 280 | 4.27 | 0.64 | <0.001 | [20] |
| **29- Gulf of Tonkin, Vietnam** | 295 | 80 | 215 | 0.06 | 0.66 | 523 | 3.04 | 0.59 | <0.001 | [20] |
| **30- Coyuca Lagoon, Mexico** | 44 | 10 | 34 | 0.34 | 0.43 | 104 | 30.59 | 0.27 | 0.76 | [21] |

*Abbreviations: S* = Species richness in the network, *H* = Number of host species in the network, *Pa* = number of parasite species in the network, *La* = proportion of parasite species at larval stage in the network, *Ad* = proportion of parasite species at adult stage in the network, *C* = connectance, *M* = modularity, *modules* = number of modules found. Note: J L Luque complemented the data used in the network 9.

***References***

1. Chemes SB, Takemoto RM (2011) Diversity of parasites from Middle Paraná system freshwater fishes, Argentina. Int J Biodivers Conserv 3: 249–266.

2. Takemoto RM, Pavanelli GC, Lizama MAP, Lacerda ACF, Yamada FH, et al. (2009) Diversity of parasites of ﬁsh from the upper Paraná River ﬂoodplain, Brazil. Braz J Biol 69: 691–705.

3. Lima Jr DP, Giacomini HC, Takemoto RM, Agostinho AA, Bini LM (2012) Patterns of interactions of a large ﬁsh–parasite network in a tropical ﬂoodplain. J Anim Ecol 81: 905–913.

4. Chinniah VC, Threlfall W (1978) Metazoan parasites of fish from the Smallwood Reservoir, Labrador, Canada. J Fish Biol 13: 203–213.

5. Arai HP, Mudry DR (1983) Protozoan and metazoan parasites of fishes from the headwaters of the Parsnip and McGregor Rivers, British Columbia: a study of possible parasite transfaunations. Can J Fish Aquat Sci 40: 1676–1684.

6. Dechtiar AO (1972) Parasites of fish from Lake of the Woods, Ontario. J Fish Res Board Can 29: 275–283.

7. Leong TS, Holmes JC (1981) Communities of metazoan parasites in open water fishes of Cold Lake, Alberta. J Fish Biol 18: 693–713.

8. Arthur JR, Margolis L, Arai HP (1976) Parasites of fishes of Aishihik and Stevens Lakes, Yukon Territory, and potential consequences of their interlake transfer through a proposed water diversion for hydroelectrical purposes. J Fish Res Board Can 33: 2489–2499.

9. Bellay S, Lima Jr. DP, Takemoto RM, Luque JL (2011) A host-endoparasite network of Neotropical marine ﬁsh: are there organizational patterns? Parasitology 138: 1945–1952.

10. Choudhury A, Hoffnagle TL, Cole RA (2004) Parasites of native and nonnative fishes of the Little Colorado River, Grand Canyon, Arizona. J Parasitol 90: 1042–1053.

11. Muzzall PM, Whelan G (2011) Parasites of fish from the Great Lakes: a synopsis and review of the literature, 1871-2010. Ann Arbor: Great Lakes Fishery Commission. 560 p. Available: www.glfc.org/pubs/SpecialPubs/2011-01.pdf. Accessed 11 December 2012.

12. Azevedo R, Abdallah V, Luque J (2010) Acanthocephala, Annelida, Arthropoda, Myxozoa, Nematoda and Platyhelminthes parasites of fishes from the Guandu river, Rio de Janeiro, Brazil. Check List 6: 659–667.

13. Morozińska-Gogol J (2007) Metazoan parasites of fish from the Łebsko Lagoon (Central Coast, Poland). Balt Coast Zone 11: 51–58.

14. Jalali B, Barzegar M (2006) Fish Parasites in Zarivar Lake. J Agric Sci Technol 8: 47–58.

15. Bensley M, Dick TA, Hudson C, Lumsden JS, Peters KK, et al. (2011) Devils Lake – Red River Basin fish parasite and pathogen project: qualitative risk assessment. International Joint Commission. 45 p. Available: http://www.ijc.org/rel/news/2011/111027_e.htm. Accessed 11 December 2012.

16. Arthur JR, Ahmed ATA (2002) Checklist of the parasites of fishes of Bangladesh. FAO Fisheries Technical Paper 369/1. Rome: FAO. 77 p. Available: http://www.fao.org/docrep/005/Y4350E/y4350e00.htm. Accessed 11 December 2012.

17. Székely C, Molnár K (1997) Preliminary survey of the parasite fauna of some important fish species in the Upper-Reservoir of the Kis-Balaton System. Parasitol Hung 29-30: 45–54.

18. Kirjušina M, Vismanis K (2007) Checklist of the parasites of fishes of Latvia. FAO Fisheries Technical Paper. No. 369/3. Rome: FAO. 106 p. Available: http://www.fao.org/docrep/010/a1078e/a1078e00.htm. Accessed 11 December 2012.

19. Violante-González J, Aguirre-Macedo ML, Mendoza-Franco EF (2007) A checklist of metazoan parasites of fish from Tres Palos Lagoon, Guerrero, Mexico. Parasitol res 102: 151–161.

20. Arthur JR, Te BQ (2006) Checklist of the parasites of fishes of Viet Nam. FAO Fisheries Technical Paper. No. 369/2. Rome: FAO. 133 p. Available: http://www.fao.org/docrep/009/a0878e/a0878e00.htm. Accessed 11 December 2012.

21. Violante-González J, Aguirre-Macedo ML (2007) A checklist of metazoan parasites of fish from Tres Palos Lagoon, Guerrero, Mexico. Parasitol Res 102: 151–161.
